# Adherence to oral antineoplastic agents, an overview and meta-analysis

**DOI:** 10.1093/oncolo/oyag168

**Published:** 2026-04-30

**Authors:** David Mourath, Helena Fohlin

**Affiliations:** Department of Oncology, Linköping University Hospital, Linköping, 58185, Sweden; Regional Cancer Center Southeast Sweden, Linköping, 58185, Sweden; Department of Biomedical and Clinical Sciences, Linköping University, Linköping, 58185, Sweden

**Keywords:** adherence, compliance, antineoplastic agents, chemotherapy, oral cancer agents, oral drug administration

## Abstract

**Background:**

The use of oral antineoplastic therapies has accelerated in recent years. Non-supervised administration of oncological therapies introduces uncertainty in regard to adherence. This review and meta-analysis reports on available literature relating to adherence, adherence-measuring methods, interventions to improve adherence, and estimates patient adherence to common oral antineoplastic therapies.

**Methods:**

A literature search was conducted in the PubMed database up to January 29, 2024 using relevant terminology for oral antineoplastic agents. Titles and abstracts of retrieved articles were screened, and full-text articles deemed of interest were reviewed. Studies suitable for meta-analysis and consistent with the analytical approach were selected based on adherence measurement methods and definitions. Random effects meta-analysis was conducted using the R package metafor and visualized with forest plots for cumulative adherence and lower-bound adherence separately.

**Results:**

We identified eight reviews and 75 original studies on adherence to non-endocrine oral antineoplastic agents, spanning the years from 1987 to 2023, with the vast majority published from 2010 onward. This review estimates the cumulative adherence to oral antineoplastic agents to be 84% (95% CI, 78–89) and lower-bound adherence as 71% (95% CI, 63–78).

**Conclusion:**

The definition of non-adherence and the measurement methods used across studies vary, rendering the comprehensive understanding of the subject challenging. This review identifies an adherence rate that deviates from full compliance across a wide variety of treatment regimens and tumor types, which could provide valuable insights for healthcare professionals in optimizing patient management.

Implications for Practice An increasing number of oncological therapies are administered orally by patients at home, rather than being given intravenously in hospitals. This introduces adherence as a potential limitation in treatment effect. By combining data from 28 different studies and over 35 000 patients, it was shown that approximately 3 out of 10 patients are at risk of significant undertreatment due to non-adherence. This should be taken into consideration in clinical practice, particularly in the event of treatment failure.

## Introduction

Oral anticancer agents have been available for use since 1957 with the approval of the alkylating agent chlorambucil.^1^ Initially, only traditional chemotherapeutic agents were available, such as cyclophosphamide, methotrexate, mercaptopurine, vinorelbine, and etoposide.^2–4^ Capecitabine entered clinical use in 1998, and the first targeted therapy, Imatinib, received FDA approval in 2001. As of writing, 87 oral antineoplastic agents, excluding endocrine therapy, are available for use in Sweden (Table S1).^5^ In 2003, the World Health Organization published a report on adherence to medication in a wide range of chronic conditions. Results varied between studies and underling disease, but adherence was found to be low, averaging 50% in developed countries.^6^ However, this report did not include any antineoplastic agents. There are several published studies on adherence to oral antineoplastic agents. These are reviewed below, together with a meta-analysis aiming to estimate real-world adherence.

Several studies in both oncology and hematology have linked low adherence to adverse outcomes.^7–10^ A recent systematic review by Lasala and Santoleri, which included 42 studies on oral antineoplastic agents, concluded that adherence appears to influence molecular response, progression-free survival, and overall survival.^11^ Similarly, low adherence has also been associated with an increased need for healthcare contacts, more frequent hospitalizations, and higher healthcare costs.^12^^,^^13^

Adherence, along with its synonym term compliance, pertains to the degree to which prescribed regimens are followed concerning timing, dosage, and frequency. The term adherence is preferred by the World Health Organization. Adherence should be differentiated from the term ‘persistence’ used to describe the sustained continuation of treatment for the prescribed duration. The definitions here laid out are applied uniformly throughout all referenced literature and are further discussed in the article authored by Cramer et al.^14^

This literature review will focus on traditional chemotherapy and targeted therapy, such as tyrosine kinase inhibitors (TKIs), in adults. Adjacent to studies on adherence levels is a growing body of work examining different interventions to increase adherence as well as factors influencing adherence. These aspects will be briefly presented and discussed.

## Methods

PubMed was searched for clinical studies in humans through January 29, 2024 using the following search terms:

“Adherence,” “Compliance,” “Oral chemotherapy,” “Oral antineoplastic therapies,” “Oral (anti-) cancer agents,” “Oral (anti-) neoplastic agents,” “Oral (anti-) cancer drugs,” “Oral (anti-) neoplastic drugs,” “Oral (anti-) cancer medicine,” “Oral (anti-) neoplastic medicine,” “Oral therapy for cancer,” and “Oral anticancer treatment.”

The PICO framework was applied to screen the results: Population = adult patients treated with non-endocrine oral antineoplastic agents; Intervention = any adherence measuring technique; Outcome = quantitative adherence outcomes. The comparison element of the PICO framework was omitted as no relevant comparison was identified. All studies concerning adherence to non-endocrine oral antineoplastic agents were eligible for inclusion in the review. Inclusion in the meta-analysis was subject to stricter criteria, which are described in detail in the meta-analysis section.

Initial screening was done by review of title and abstracts of all search results. Included articles were read in full, and publication journal, author, year, sample size, oral agent(s), study duration, adherence definition(s), adherence measurement method(s), and reported adherence extracted.

### Statistical analysis

A random-effects meta-analysis was conducted using the R package *metafor* (version 4.6.0).^15^ Pooled estimates were calculated using a random-effects model to account for between-study heterogeneity. The between-study variance (τ^2^) was estimated using restricted maximum likelihood.

For proportion data, adherence rates were transformed using the logit transformation prior to analysis to stabilize variances. Pooled estimates were subsequently back-transformed to the original proportion scale and presented as percentages with 95% confidence intervals.

Statistical heterogeneity was performed using Cochran’s Q test and quantified with the I^2^ statistic. Contribution of subgroup variables to heterogeneity was assessed using the Omnibus Q test for moderators (QM). Funnel plots were generated and assessed for asymmetry using Egger’s test.

All tests were two-sided, and a *P*-value < .05 was considered statistically significant. The statistical analyses were performed using R version 4.3.1 (2023-06-16 ucrt).^16^

## Results

**Figure 1.**
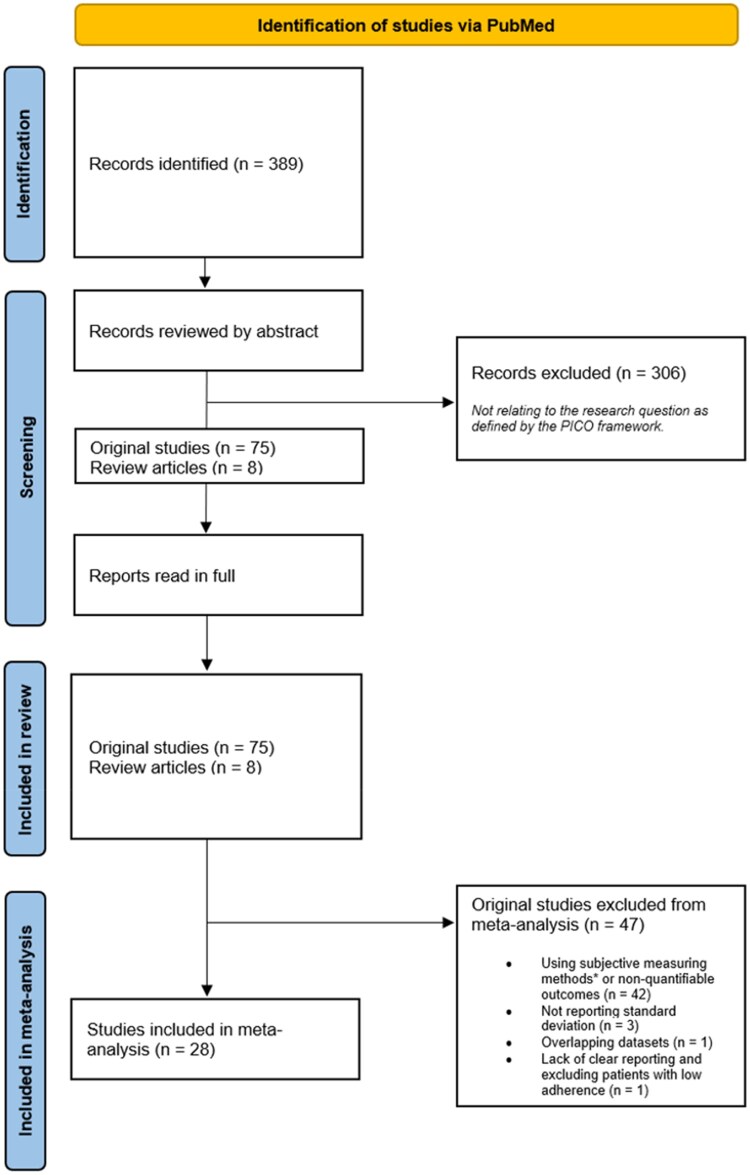
PRISMA diagram describing screening and inclusion. *Subjective measuring methods include self-report and pill-counts.

Eight reviews on the topic were identified. One recent and comprehensive review is written by Greer et al.^17^ and includes 51 studies, with 19 specifically evaluating non-hormonal agents and spanned studies up until 2015. Special emphasis was placed on updating the knowledge gained from this review. This search resulted in the identification of 389 studies through the specified search criteria. A total of 75 original studies on adherence to non-endocrine oral antineoplastic agents are included in this review spanning the years from 1987 to 2023. A flow diagram depicting the selection process can be viewed in Figure 1.

There exists an inconsistency in certain fundamental concepts across studies, rendering the comprehensive understanding of the subject challenging. These are (1) difference in adherence measuring methods and (2) what level of adherence should be considered non-adherence. These differences will be presented below to aid in data interpretation.

### Difference in adherence measuring methods

Five fundamentally different methods of measuring adherence are present in the literature. These are:

- Measurement of the level of therapeutic agent or metabolite in blood or urine
- Medical event monitoring systems (MEMS)
- Adherence calculated through prescription refills as either medication possession ratio (MPR) or proportion of days covered (PDC)
- Pill-counts by medical staff
- Patient self-report including patient questionnaires such as “Morisky Medication Adherence Scale” (MMAS), “Medication Adherence Report Scale” (MARS), “Basel Assessment of Adherence Scale” (BAAS), “Brief Adherence Rating Scale” (BARS), or Visual Analog Scale (VAS) among others. Several studies use proprietary scales unique to each study.

Each of these methods comes with their own strengths and drawbacks. We propose the division of adherence measuring methods into 2 sections: objective and subjective. This is also suggested by the World Health Organization.^6^ Objective measurement methods for assessing medication adherence include MEMS, MPR, PDC, and measurement of the level of medicine or metabolite in blood or urine.

**Blood/urine drug levels** present an intriguing prospect for adherence studies due to their inherent objectivity and capability to gauge both ingestion and pharmacokinetics. However, there are notable drawbacks to this approach. Variability in metabolic products among patients and the inability to evaluate the timing of doses pose significant challenges.^18^^,^^19^ In addition, there is a hypothesized risk of additional dosing, “white-coat compliance,” before a doctor’s visit, leading to artificially elevated outcomes.^18^^,^^19^ The associated high costs and the necessity for specialized equipment further diminish its suitability for adherence measurement studies.

**MEMS** encompass devices with microcircuitry and sensors designed to record the number of medication doses taken. The most employed form involves caps on medication bottles that store the time, date, and number of cap removals. This method offers several strengths, including ease of deployment, prolonged data collection with minimal patient intrusion, and the ability to quantify adherence. However, it is important to note that MEMS only capture the surrogate marker of cap openings, not the actual ingestion of the therapeutic agent.

**MPR and PDC** are database methods using records on prescribed medications to calculate the quota of days patients would have been able to ingest medication derived from their medication refills. MPR and PDC use slightly different calculations that are described in great detail by Asamoa-Boaheng et al.^20^ However, distinguishing between them were not deemed necessary for the purposes of this review. Utilizing either MPR or PDC enables researchers to retrospectively collect data from very large patient populations over extended periods, with minimal interference to the patient. One major drawback, as with MEMS, is that prescription refill is not equivalent to ingested agent. Adding to this is the risk of non-persistence being calculated as non-adherence.

**Pill-counts** as a measuring method are quite homogeneous between studies and consist of the nurse or doctor counting remaining pills in tablet containers and are often classified as an objective measure of adherence. However, we would argue that pill counting differs from other objective methods in that it is more susceptible to misreporting.^19^ Unlike MEMS, which records exact dosing times and opening events throughout the observation period, pill-counts provide only a cross-sectional estimate at the end of the period and do not capture timing of medication use. In contrast to measures such as MPR or PDC, which are calculated retrospectively, pill-counts are typically performed with the patient’s awareness, creating the possibility of behavioral adaptation, including so called “pill-dumping.”^21^ This interpretation is supported by studies in which pill-counts have indicated higher adherence than MEMS within the same population.^22^^,^^23^ In light of this, we chose to exclude studies that relied exclusively on pill counting from the planned meta-analysis.

Subjective measuring methods include all different types of self-reports. **Self-report** is a convenient and often used method of adherence measurement. Its strength lies in it being the only measuring tool able to capture true patient behavior of ingestion of the agent combined with its ease of use and low cost.^24^ Drawbacks include the potential for recall bias and the necessity for tool validation. It is worth noting that the article validating MARS for use in patients taking anti-hypertensive medications has been retracted due to concerns regarding the statistical analysis used.^25^ The use of this reporting method in studies may introduce uncertainties and caution may be advised in interpreting results from this and other less validated scales. Several studies examining the correlation between self-report methods and different objective measurements show substantial differences in reported adherence,^22^^,^^23^^,^^26–33^ as do two review articles.^17^^,^^19^ This is outlined in the discussion below.

### Definition of non-adherence

Data on adherence is calculated and presented in 4 different ways:

1. The percentage of pills taken out of the total amount planned for the treatment period. This is often acquired in studies using MEMS or MPR/PDC. This way of reporting will in this paper be referred to as “cumulative adherence.”
2. The proportion of patients taking at least a certain percentage of doses. The lower bound is often set as 80% or 90%, although 70% and 85% are occasionally used, for patients to be considered adherent. This way of reporting will in this paper be referred to as “lower-bound adherence.”
3. “Absolute adherence” in which a patient is marked as a non-adherer if they fail to comply to the regimen 100%. Studies using self-report and doctor interviews often present their data in this way. Studies measuring drug metabolites in urine or blood often present their data in a yes/no fashion closely resembling “absolute adherence.” Studies using MPR/PDC or MEMS seldom present their data this way.
4. Some studies present their adherence data as a score on one of several “standardized adherence questionnaires” ex MARS/BAAS/MMAS. A large proportion of these use proprietary scales unique to each study. This way of reporting will in this paper be referred to as “questionnaire-adherence.”

Most studies are in agreement regarding adherence < 80% being the threshold for non-adherence when cumulative reporting is not used.

### Reviews

Eight reviews concerning oral antineoplastic agents were identified, and their key characteristics are summarized in Table 1.

**Table 1.** Characteristics of review articles on rates of adherence, interventions and influencing factors.

Author, Year	Number of included studies (cancer types)	The article’s judgment of included studies	Methods and definitions	Conclusion (total number of included patients)
**Reviews concerning adherence**				
**Greer et al.,^17^ 2016**	19 concerning non-endocrine therapy. (Breast, colorectal, liver, GIST, CML)	The majority of included studies had poor methodological quality.	MEMS, MPR, PDC, pill-counts, self-report, chart review. A variety of different adherence definitions were used.	“Adherence estimates ranged widely across studies from 46% to 100%.” (*n* = 5609)
**Ruddy et al., ^34^ 2009**	8 concerning non-endocrine therapy. (Breast, lung, ovarian, colon, lymphoma, MDS, undefined hematological)	NR	Serum or urine analysis, MEMS, MPR, pill-counts, self-report. A variety of different adherence definitions were used.	“Adherence and persistence rates ranged from 16% to 100% with different therapies and different methods of measurement.” (*n* = 541)
**Partrige et al.,^18^ 2002**	5 concerning non-endocrine therapy in adults, all of which were included in the review by Ruddy et al. (34). (Breast, lung, ovarian, lymphoma, undefined hematological)	NR	Serum concentration, MEMS, pill-counts, self-report. A variety of different adherence definitions were used.	“Adherence rates ranging from less than 20% to 100% have been reported…” (*n* = 229)
**Barillet et al., ^24^ 2015**	29 concerning non-endocrine therapy. (Breast, colorectal, CML, GIST, lymphoma, lung, ovarian, MDS)	Major differences in measuring methods and definitions of adherence were noted, complicating comparisons across studies.	Urine analysis, MEMS, MPR, pill-count, self-report. A variety of different adherence definitions were used.	“Adherence rates were found to vary from 14% to 100%.” (*n* = NR)
**Mislang et al., ^35^ 2017** ^a^	9 concerning non-endocrine therapy in patients >65 years of age. (Breast, colorectal, gastrointestinal, lung, CML, GIST, prostate, mixed)	NR	Blood tests, MEMS, MPR, pill-count, self-report. A variety of different adherence definitions were used.	Reported adherence ranges were 29.2% to 96.8%. (*n* = 4108)
**Reviews concerning interventions**				
**Dang et al.,^36^ 2022**	Overview of reviews, 29 included reviews (Mixed solid and hematological cancers.)	28 of the included studies were of low or very low quality.	Serum or urine analysis, MEMS, MPR, pill-counts, self-report. A variety of different adherence definitions were used.	Simple interventions such as education or reminders was not able to improve adherence. However, interventions combining multiple strategies were more likely to show an effect.
**Rosenberg et al.,^37^ 2020**	9 randomized controlled trials concerning non-endocrine therapy. (Mixed solid and hematological cancers.)	Generally small sample sizes.	Serum concentration, MEMS, MPR, pill-counts, self-report. A variety of different adherence definitions were used.	3 out of 9 studies showed a clinical meaningful effect on adherence. 1 of which did not report any *P*-value, the other 2 used self-report measurement methods.
**Reviews concerning factors influencing adherence**				
**Mathes et al.,^38^ 2014**	22 studies and 23 publications concerning both endocrine and non-endocrine therapies.	NR	Serum or urine analysis, MEMS, MPR, pill-counts, self-report. A variety of different adherence definitions were used.	Possible influencing factors: Age, social support, marriage, dose, depression, medication cost, polypharmacy, socioeconomic status.
**Mislang et al.,^35^ 2017** ^a^	26 studies including both endocrine and non-endocrine therapies.	NR	Blood tests, MEMS, MPR, pill-count, self-report. A variety of different adherence definitions were used.	Only included articles investigating adherence rates of patients >65 years of age. Possible influencing factors: Cognitive impairment, multimorbidity, polypharmacy, poor doctor communication skills and medication cost.

Abbreviations: CML = chronic myeloid leukemia, MDS = myelodysplastic syndrome, GIST = gastrointestinal stromal tumor, NR = not reported.

a Included in both adherence and adherence contributing factors.

The real-world implication of the adherence levels reported in these reviews is not immediately clear due to all of them combining different definitions of adherence, which is the nature of the source material. The rather often used definition of absolute adherence combined with a variety of different questionnaires and scales muddles the results. All reviews referenced studies with adherence levels very close to and sometimes exceeding 100%.^17^^,^^31^ However, these cases were in the minority, and non-adherence is consistently observed across all cancer types and oral antineoplastic drugs. Several reviews underscore this as a significant challenge in cancer treatment.^24^^,^^35^

### Original studies on adherence

Seventy-five original studies were examined and can be viewed in Table 2. In studies assessing the impact of adherence-altering interventions, only the adherence rates of the control group or adherence at baseline are reported.

**Table 2.** Characteristics of original studies on rates of adherence.

Author, Year	Oral therapy	Number of patients	Study duration	Adherence definition	Measurement method	Adherence
**Chronic myeloid leukemia (*n* = 15)**						
**Darkow et al.,^12^ 2007**	Imatinib	267	52 weeks	Cumulative & Lower-bound (90%)	MPR	77% 54%
**Kiguchi et al.,^39^ 2009**	Imatinib	52	Unclear	Cumulative	MPR	93-98%
**Noens et al.,^31^ 2009**	Imatinib	169	13 weeks	Mixed	Pill counts, Self-report (BAAS, VAS)	Pill-counts: 90% BAAS: 68% VAS: 95%
**Wu et al.,^13^ 2010**	Imatinib	592	52 weeks	Cumulative & Lower-bound (85%)	MPR	79% 59%
**Wu et al.,^40^ 2010**	Dasatinib, Nilotinib	521	26 weeks	Cumulative	PDC	Dasatinib: 69% Nilotinib: 79%
**Ganesan et al.,^7^ 2011**	Imatinib	516	170 weeks	Absolute	Patient records	71%
**Jönsson et al.,^41^ 2011**	Imatinib	38	Unclear	Questionnaire	Self-report (MMAS)	97%
**Guérin et al.,^42^ 2012**	Dasatinib, Nilotinib	878	52 weeks	Cumulative	PDC	Dasatinib: 69% Nilotinib: 75%
**Efficace et al.,^43^ 2012**	Imatinib	413	>156 weeks	Questionnaire	Self-report (MMAS)	53%
**Santoleri et al.,^44^ 2013**	Imatinib, Dasatinib, Nilotinib	91	>52 weeks	Cumulative	MPR	Imatinib: 83% Dasatinib: 85% Nilotinib: 93%
**Trivedi et al.,^45^ 2014**	Dasatinib, Nilotinib	377	52 weeks	Cumulative & Lower-bound (85%)	PDC MPR	Dasatinib: 79% Nilotinib: 77% Dasatinib: 73% Nilotinib: 63%
**Anderson et al.,^46^ 2015**	Imatinib, Dasatinib, Nilotinib	124	78 weeks	Lower-bound (90%)	MPR	69%
**Kekäle et al.,^47^ 2016**	Imatinib	86	39 weeks	Questionnaire	Self-report (MMAS)	21%
**Hefner et al.,^48^ 2017**	TKIs	35	Unclear	Questionnaire	Self-report (BAAS)	51%
**Dennison et al.,^49^ 2021**	Imatinib, Dasatinib, Nilotinib, Bosutinib	70	>13 weeks	Questionnaire	Self-report (MMAS)	60%
**Chronic lymphocytic leukemia**						
**Ochagavía et al.,^33^ 2023**	Ibrutinib, Venetoclax	23	13 weeks	Lower-bound (90%)	Pill-counts, Self-report (MMAS)	100%
**Lymphoma**						
**Lee et al.,^50^ 1992**	Chlorambucil, Prednisolone, Dexamethasone	21	2 weeks	Cumulative	MEMS	100%
**Multiple myeloma (*n* = 2)**						
**Arber et al.,^51^ 2017**	Cyclophosphamide, Thalidomide, Dexamethasone	64	NR	Absolute	Self-report	92%
**Gatwood et al.,^52^ 2022**	Thalidomide, Lenalidomide, Pomalidomide	Commercial: 585 Medicare: 1865^a^	52 weeks	Cumulative & Lower-bound (80%)	PDC	Commercial: 58% Medicare: 65% Commercial: 24% Medicare: 42%
**Myelodysplastic syndrome**						
**Klein et al.,^53^ 2006**	Topotecan	90	3 weeks	Absolute	Concentration measurements, MEMS	90%
**Other hematological malignancies (*n* = 2)**						
**Levine et al.,^26^ 1987** **(Undefined malignancy)**	Prednisolone, Allopurinol	108	26 weeks	Absolute	Concentration measurements	Prednisolone: 26.8% Allopurinol : 16.8%
**Larizza et al.,^54^ 2006** **(KML and MM)**	Imatinib, Thalidomide	24	Unclear	Absolute	Self-report (MMAS)	67%
**Breast cancer (*n* = 6)**						
**Lebovits et al.,^55^ 1990**	Cyclophosphamide, Prednisolone	51	26 weeks	Lower-bound (90%)	Self-report	Cyclophosphamide: 53% Prednisolone: 57%
**Mayer et al.,^56^ 2009**	Capecitabin, Gefitinib	13	6 weeks	Cumulative	MEMS	98%
**Partridge et al.,^57^ 2010**	Capecitabine	150	18 weeks	Cumulative & Lower-bound (80%)	MEMS	78% 75%
**Ruddy et al.,^58^ 2012**	Cyclophosphamide	133	24 weeks	Cumulative	Self-report (Calander)	97% 5% below 80%
**Komatsu et al.,^59^ 2020**	Capecitabine, Lapatinib, Tegafur, Gimeracil, Oteracil	59	13 weeks	Lower-bound (90%)	MPR	92%
**Engel-Nitz et al.,^60^ 2023**	Palbociclib	1066	26 weeks	Cumulative & Lower-bound (80%)	MPR	88% 80%
**GIST (*n* = 2)**						
**Mazzeo et al.,^61^ 2011**	Imatinib	28	13 weeks	Questionnaire	Self-report (BAAS, VAS)	BAAS: 76% VAS: 95%-97%
**Wang et al.,^62^ 2019**	Imatinib	158	≥4 weeks	Questionnaire	Self-report (MMAS)	42%
**Colorectal cancer (*n* = 4)**						
**Sadahiro et al.,^63^ 2000**	Urasil-tegafur	87	52 weeks	Questionnaire	Concentration measurements, Self-report	91%
**Kawakami et al.,^64^ 2015**	Capecitabine	236	16 weeks	NR	Self-report	98%
**Font et al.,^29^ 2017**	Capecitabine	119	6 weeks	Lower-bound (80%)	Pill-counts, Self-report, Doctors report	Pill-counts: 67% Self-report: 83% Doctors report: 100%
**Jiang et al.,^65^ 2019**	Capecitabine	50	6 weeks	Cumulative	MEMS	85%
**Gastric cancer**						
**Kimura et al.,^66^ 2023**	Capecitabine	64	24 weeks	Absolute	Pill-counts	42%
**Renal cell carcinoma**						
**Dinan et al., ^9^ 2022**	Sorafenib, Pazopanib, Sunitinib, Everolimus, Axitinib	905	13 weeks	Cumulative & Lower-bound (80%)	PDC	69% 49%
**Hepatocellular carcinoma**						
**Mallick et al.,^67^ 2013**	Sorafenib	1127	17 weeks	Cumulative & Lower-bound (80%)	PDC	89% 79%
**Lung cancer (*n* = 2)**						
**Lee et al.,^68^ 1993**	Etoposide	12	6 weeks	Cumulative	MEMS	93%
**Hess et al.,^69^ 2017**	Erlotinib	1088	29 weeks	Lower-bound (80%)	MPR	88%
**Gynecologic cancer** **(*n* = 3)**						
**Lee et al.,^70^ 1996**	Altretamine	11	4 weeks	Cumulative	MEMS	97%
**Watson et al.,^71^ 2020**	Letrozole, PARPi, Tamoxifen, Megace, Trametinib, Exemestane, Lenvatinib	100	≥4 weeks	Absolute	Self-report	54%
**Moss et al.,^72^ 2021**	PARP-inhibitors	151	22 weeks	Lower-bound (80%)	PDC	79%
**Mixed cancers (*n* = 33)**						
**Macintosh et al.,^73^ 2007**	Capecitabine	18	2 weeks	Absolute	Pill-counts, Self-report	84%
**Marques et al.,^74^ 2008**	TKIs, Capecitabine, Temozolomide and endocrine therapy	61	37 weeks	Questionnaire	Self-report (MMAS)	72%
**Decker et al.,^75^ 2009**	TKIs, Capecitabine, Cyclophosphamide, Methotrexate	30	8 weeks	Absolut	Pill-counts, Self-report	77%
**Saratsiotou et al.,^76^ 2011**	TKIs, Capecitabine, Vinorelbine	99	Unclear	Questionnaire	Self-report	79%
**Winterhalder et al.,^77^ 2011**	Capecitabine	177	Unclear	Questionnaire	Self-report	91%
**Thivat et al.,^78^ 2012**	Capecitabine	14	26 weeks	Lower-bound (80%)	MEMS	89%
**Bhattacharya et al.,^79^ 2012**	Capecitabine	43	17 weeks	Questionnaire	Self-report (MARS)	77%
**Spoelstra et al.,^80^ 2013**	NR	100	8 weeks	Lower-bound (80%)	MPR	67%
**Schneider et al.,^27^ 2014**	TKI, Capecitabine and endocrine therapy (AI, TAM)	20	17 weeks	Cumulative	MPR, Self-report	69%
**Figueiredo et al.,^81^ 2014**	Capecitabine	30	8-12 weeks	Absolute	Pill-counts	88%-96% (Depending on cancer type)
**Ramesh et al.,^82^ 2015**	NR	60	NR	Questionnaire	Self-report (MARS)	83%
**Spoelstra et al.,^83^ 2015**	NR	37	8 weeks	Questionnaire	Self-report	55%
**Kimura et al.,^84^ 2015**	Capecitabine, S-1	172	20 weeks	Absolute	Self-report	Capecitabine: 61% S-1: 65%
**Barthélémy et al.,^85^ 2015**	TKIs, Endocrine therapy, unspecified chemotherapy.	201	48 weeks	Questionnaire	Self-report	71%
**Feiten et al.,^86^ 2016**	Capecitabine, Pazopanib, Temozolomide, endocrine therapy and “others”	109	26 weeks	Lower-bound (80%)	MPR	94%
**Spoelstra et al.,^87^ 2016**	NR	24	9 weeks	Absolute	Pill-counts, Self-report	79%
**Jacobs et al.,^88^ 2017**	TKIs, Capecitabine and endocrine therapy	82	13 weeks	Cumulative & Lower-bound (90%)	MEMS	89% 72%
**Stokes et al.,^89^ 2017**	Imatinib, Erlotinib, Capecitabine	22219	23 weeks	Lower-bound (80%)	MPR	56%
**Kovacic et al.,^90^ 2017**	Capecitabine	623	Unclear	Absolute	MPR	90%
**Fernández-Ribeiro et al.,^91^ 2017**	Capecitabine	111	30 weeks	Absolute	Pill-counts, Self-report (MMAS)	78%
**Hirao et al.,^92^ 2017**	TKIs, Capecitabine, Tegafur	117	47 weeks	Questionnaire	Self-report	56%
**Salgado et al.,^93^ 2017**	Imatinib, Lenalidomide, Capecitabine, Hydroxyurea, Ibrutinib, Abiraterone, Crizotinib, Erlotinib, Temozolomide and “others”	125	≥4 weeks	Questionnaire	Self-report	70%
**Morgan et al.,^94^ 2018**	TKIs, Capecitabine, Everolimus, Temozolomide and endocrine therapy	66	13 weeks	Cumulative	MPR	92%
**Middendorff et al.,^95^ 2018**	TKIs, Capecitabine, Everolimus and endocrine therapy	40	26 weeks	Cumulative & Lower-bound (80%)	MPR	92% 85%
**Muluneh et al.,^96^ 2018**	NR	93	Unclear	Questionnaire	Self-report	70%
**Hefner et al.,^97^ 2018**	Capecitabine	64	30 weeks	Questionnaire	Self-report (MARS)	80%
**Jacobs et al.,^98^ 2019**	TKIs, Capecitabine, Temozolomide, Everolimus, Palbociclib and endocrine therapy	181	2 weeks	Cumulative & Lower-bound (80%)	MEMS	85% 83%
**Krikorian et al.,^99^ 2019**	NR. Included capecitabine and endocrine therapy.	Nurse-led: 99 Pharmacy-led: 101	4 weeks	Cumulative	MPR	Nurse-led: 96% Pharmacy-led: 99%
**Bouleftour et al.,^100^ 2021**	TKIs, Endocrine therapy, others.	183	24 weeks	Questionnaire	Self-report (MMAS)	79%
**Skrabal et al.,^101^ 2022**	TKIs, Capecitabine, Palbociclib, Ribociclib, Temozolomide, Hydroxyurea	19	10 weeks	Lower-bound (80%)	MEMS	89%
**Feral et al.,^102^ 2022**	TKIs, PARP-inhibitors, Capecitabine, Everolimus, Palbociclib, Temozolomide, Vinorelbine.	64	9 weeks	Cumulative	MPR	94%
**Chen et al.,^103^ 2022**	Capecitabine, Tegafur, “others”.	151	3 weeks	Questionnaire	Self-report (MMAS)	82%
**Melis et al.,^30^ 2023**	NR. Included endocrine therapy.	124	26 weeks	Cumulative & Lower-bound (80%)	MPR	79% 65%

‘Unclear’ indicates that study duration varied between participants and no mean duration was reported.

Abbreviations: KML = chronic myeloid leukemia. MM = multiple myeloma. GIST = gastrointestinal stromal tumor. TKI = tyrosine kinase inhibitors. AI = aromatase inhibitors. TAM = Tamoxifen. NR = not reported.

a Medicare refers to patients receiving American federal health insurance in contrast to commercially insured patients.

Studies exhibited considerable variability in measurement methodologies, definitions, and duration. While some reported excellent adherence, an even greater proportion documented suboptimal and occasionally remarkably low adherence rates.

### Interventions to increase adherence

Two reviews examined interventions to increase adherence. Rosenberg et al.^37^ conducted an analysis of nine randomized controlled trials involving adults undergoing non-endocrine therapy. Only three trials demonstrated an effect of applied interventions. The largest effect was seen by Kekäle et al.^47^ using multi-modal interventions in a population with exceptionally low baseline adherence (21%). Dang et al.^36^ conducted an overview of reviews, evaluating 29 reviews on medication adherence interventions. The overall quality of included studies was deemed as low. Simple interventions such as patient education or reminders were not able to improve adherence while interventions that combined multiple strategies were more likely to show an effect.

### Factors influencing adherence

Two reviews concerning contributing factors of adherence to oral antineoplastic agents were identified. In 2014, Mathes et al.^38^ authored a systematic review examining different factors influencing oncological patients’ adherence rates in 23 publications spanning both endocrine and non-endocrine treatment. Good social support showed a strong positive effect on adherence. Depression, high out-of-pocket cost, polypharmacy as well as low, or very high, age contributed to a lower level of adherence.

In the review article authored by Mislang et al.^35^ in 2017, adherence, and its contributing factors, to oral antineoplastic agents in the elderly (≥65 years of age) were examined throughout 26 original studies. Highlighted is that doctors with poor communication skills had a 19% increased likelihood of patient non-adherence. Additionally, cognitive impairment, multi-morbidity, and polypharmacy were identified as risk factors.

Of note is that side effects are not listed as a contributor to non-adherence in either review. Mathes et al.^38^ included 5 studies on the topic with conflicting outcomes. An original study by Anderson et al.^46^ examined adherence to imatinib, dasatinib, and nilotinib in 124 patients and found no correlation between side effects and adherence. According to Ruddy et al.,^34^ it is uncertain how side effects influence adherence and that the presence of side effect indicates efficiency and could even increase adherence to the therapy.

### Meta-analysis

In an endeavor to enhance clarity in the field of adherence to oral antineoplastic agents, studies utilizing objective measurement methods and employing quantitative reporting approaches, specifically cumulative and lower-bound adherence, were selected for meta-analysis (see Table 3). Particular emphasis was placed on including studies that used measurement methods with the lowest susceptibility to manipulation and reported outcome measures of clear clinical relevance. As noted above, pill-counts and questionnaires were considered to carry a greater risk of artificially inflated adherence estimates than MPR/PDC and MEMS. Therefore, only the latter were included in the meta-analysis. Similarly, studies reporting absolute adherence outcomes, including drug concentration measurements, were not included.

**Table 3.** Studies using objective adherence measurement methods and reporting cumulative or lower-bound rates of adherence.

Author, Year	Number of patients	Adherence definition	Measurement method	Adherence
**Studies reporting cumulative adherence (*n* = 24)**				
**Lee et al.,^50^ 1992**	21	Cumulative	MEMS	100% (SD 20.6)
**Lee et al.,^68^ 1993**	12	Cumulative	MEMS	93% (SD 12)
**Lee et al.,^70^ 1996**	11	Cumulative	MEMS	97% (SD 6.9)
**Darkow et al.,^12^ 2007**	267	Cumulative	MPR	77% (SD 27.5)
**Mayer et al.,^56^ 2009** ^a^	13	Cumulative	MEMS	98%
**Partridge et al.,^57^ 2010** ^a^	150	Cumulative	MEMS	78%
**Wu et al.,^13^ 2010**	592	Cumulative	MPR	79% (SD 30)
**Wu et al.,^40^ 2010** ^b^	521	Cumulative	PDC	Dasatinib: 69% (SD 28) Nilotinib: 79% (SD 23)
**Guérin et al.,^42^ 2012**	878	Cumulative	PDC	Dasatinib: 70% (SD 28.3) Nilotinib: 76% (SD 24.3)
**Santoleri et al.,^44^ 2013** ^a^	91	Cumulative	MPR	Imatinib: 83% Dasatinib: 85% Nilotinib: 93%
**Mallick et al.,^67^ 2013** ^a^	1127	Cumulative	PDC	89%
**Schneider et al.,^27^ 2014** ^a^	20	Cumulative	MPR, Self-report	69%
**Trivedi et al.,^45^ 2014**	377	Cumulative	PDC	Dasatinib: 79% (SD 26) Nilotinib: 77% (SD 32)
**Jacobs et al.,^88^ 2017**	82	Cumulative	MEMS	89% (SD 19.1)
**Morgan et al.,^94^ 2018**	66	Cumulative	MPR	92% (SD 10)
**Middendorff et al.,^95^ 2018**	40	Cumulative	MPR	92% (SD 12.3)
**Jacobs et al.,^98^ 2019**	181	Cumulative	MEMS	85% (SD 28)
**Jiang et al.,^65^ 2019**	50	Cumulative	MEMS	85% (SD 14.1)
**Krikorian et al.,^99^ 2019**	Nurse-led: 99 Pharmacy-led: 101	Cumulative	MPR	Nurse-led: 96% (SD 13.5) Pharmacy-led: 99% (SD 7)
**Feral et al.,^102^ 2022**	64	Cumulative	MPR	94% (SD 16)
**Gatwood et al.,^52^ 2022**	Commercial: 585 Medicare: 1865	Cumulative	PDC	Commercial: 58% (SD 24) Medicare: 65% (SD 27)
**Dinan et al.,^9^ 2022**	905	Cumulative	PDC	69% (SD 29)
**Engel-Nitz et al.,^60^ 2023**	1066	Cumulative	MPR	88% (SD 16)
**Melis et al.,^30^ 2023** ^a^	124	Cumulative	MPR	79%
**Studies reporting lower-bound adherence (*n* = 20)**				
**Darkow et al.,^12^ 2007**	267	Lower-bound (90%)	MPR	54%
**Partridge et al.,^57^ 2010**	150	Lower-bound (80%)	MEMS	75%
**Wu et al.,^13^ 2010**	592	Lower-bound (85%)	MPR	59%
**Spoelstra et al.,^80^ 2013**	100	Lower-bound (80%)	MPR	67%
**Mallick et al.,^67^ 2013**	1127	Lower-bound (80%)	PDC	79%
**Trivedi et al.,^45^ 2014**	377	Lower-bound (85%)	MPR	Dasatinib: 73% Nilotinib: 63%
**Anderson et al.,^46^ 2015**	124	Lower-bound (90%)	MPR	69%
**Feiten et al.,^86^ 2016**	109	Lower-bound (80%)	MPR	94%
**Jacobs et al.,^88^ 2017**	82	Lower-bound (90%)	MEMS	72%
**Hess et al.,^69^ 2017**	1088	Lower-bound (80%)	MPR	88%
**Stokes et al.,^89^ 2017**	22219	Lower-bound (80%)	MPR	56%
**Middendorff et al.,^95^ 2018**	40	Lower-bound (80%)	MPR	85%
**Jacobs et al.,^98^ 2019**	181	Lower-bound (80%)	MEMS	83%
**Komatsu et al.,^59^ 2020**	59	Lower-bound (90%)	MPR	92%
**Moss et al.,^72^ 2021**	151	Lower-bound (80%)	PDC	79%
**Dinan et al.,^9^ 2022**	905	Lower-bound (80%)	PDC	49%
**Skrabal et al.,^101^ 2022**	19	Lower-bound (80%)	MEMS	89%
**Gatwood et al.,^52^ 2022**	Commercial: 585 Medicare: 1865	Lower-bound (80%)	PDC	Commercial: 24% Medicare: 42%
**Engel-Nitz et al.,^60^ 2023**	1066	Lower-bound (80%)	MPR	80%
**Melis et al.,^30^ 2023**	124	Lower-bound (80%)	MPR	65%

Abbreviation: SD = standard deviation.

a Studies excluded from meta-analysis due to no standard deviation being reported.

b Excluded from meta-analysis due to data set overlap with Guérin et al.

Random effects meta-analysis was conducted. The model assumes that the true effect size varies between studies due to differences in study populations, methodologies, or other factors so that the studies estimate different but related true effects.

Data from 28 original studies employing MEMS, MPR, or PDC as measurement methods were used. Outcomes are separated into

1. cumulative adherence, defined as the percentage of prescribed doses taken and
2. lower-bound adherence, defined as the proportion of patients who ingest at least a predetermined minimum fraction of their prescribed doses.

**Figure 2.**
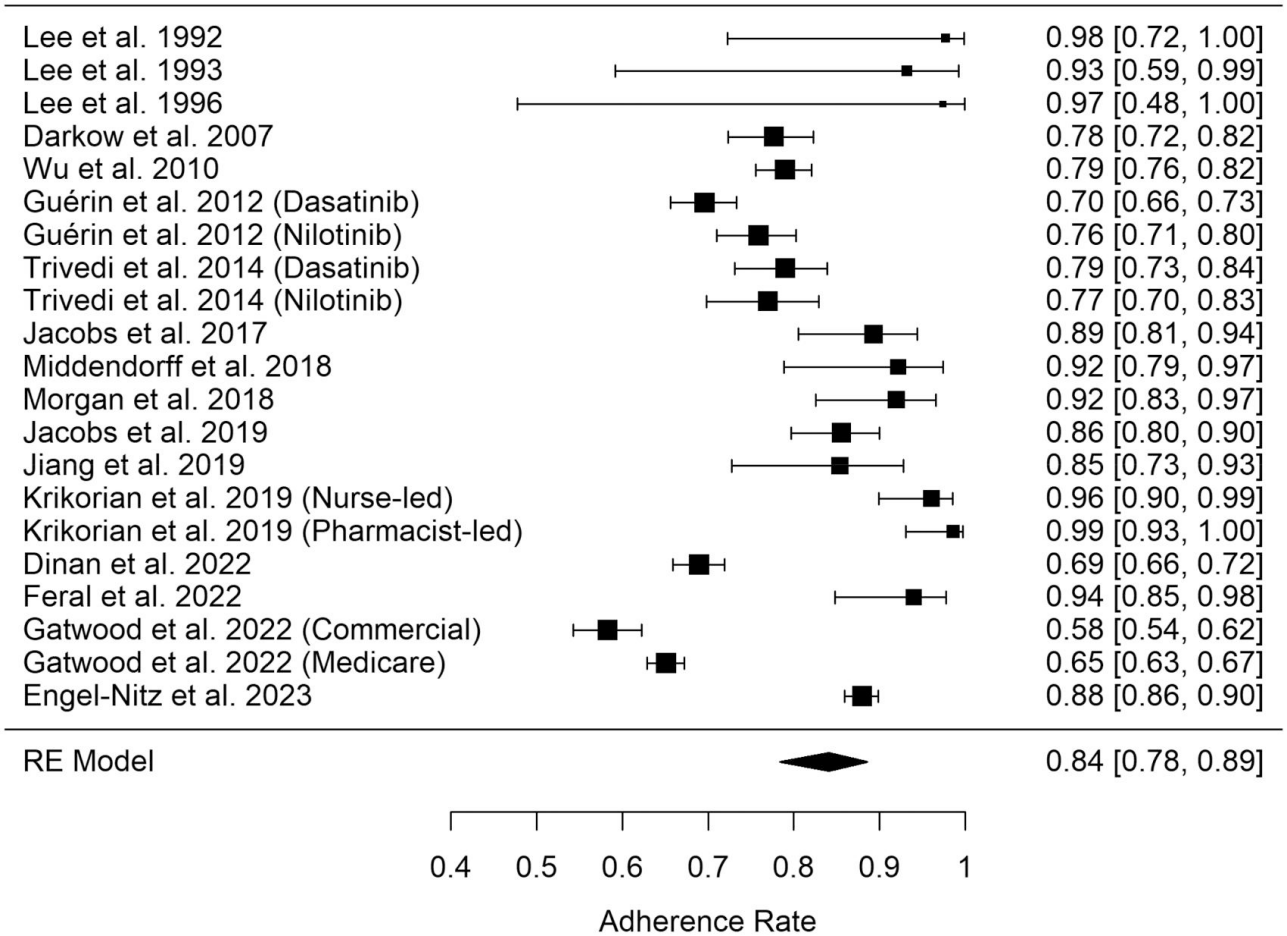
Forest plot depicting adherence and 95% CI to oral anti-neoplastic therapies derived from studies reporting cumulative adherence. RE = Random effects model.

**Figure 3.**
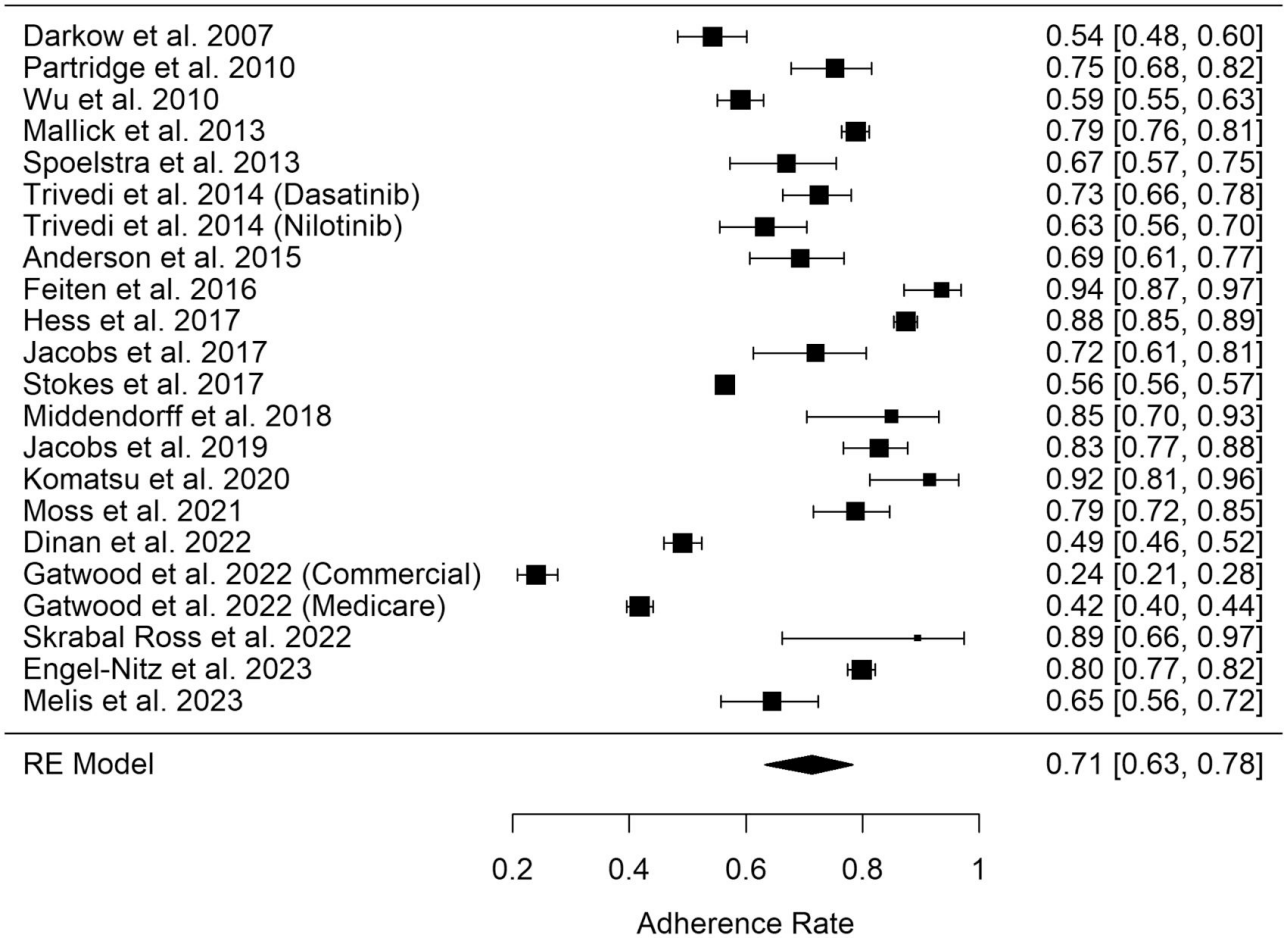
Forest plot depicting adherence and 95% CI to oral anti-neoplastic therapies derived from studies reporting lower-bound adherence. RE = Random effects model.

Some studies report both kinds of outcomes. The cumulative portion of the meta-analysis included 17 studies, 21 different cohorts, and 7 262 patients and showed a pooled adherence rate of 84% (95% CI, 78–89), as seen in Figure 2. The lower-bound portion of the meta-analysis included 20 studies, 22 different cohorts, and 31 230 patients and showed a pooled estimate of 71% (95% CI, 63–78), as seen in Figure 3. There was no significant difference in adherence, regardless of whether the lower-bound cut-off threshold was set at 80%, 85%, or 90% (*P* = .83).

Our objective was to provide insight into the real-world extent of adherence to antineoplastic therapies in a way that could support healthcare professionals in clinical practice. The high level of heterogeneity of published studies, both in methodology and results, and existing reviews reporting very wide adherence ranges (see Table 1) made the available literature suboptimal in this regard. This variability is also reflected in the meta-analysis, where Cochran’s Q test for heterogeneity revealed substantial variability between studies for both cumulative adherence (*P* < .0001) and lower-bound adherence (*P* < .0001). The proportion of total variability attributable to heterogeneity rather than chance (I^2^) was 97.1% and 99.1%, respectively, and the between-study variance (τ^2^) was estimated at 0.62 and 0.74.

Egger’s test for asymmetry was significant for cumulative adherence (*P* = .002), indicating possible publication bias or small-study effects (Figure S1). For lower-bound adherence, the asymmetry was less evident (*P* = .07) (Figure S2). Combining these diverse studies makes the results less robust, and the data should be interpreted with caution. Despite this, our analysis provide evidence that adherence to oral oncological therapy deviates substantially from 100%.

Kiguchi et al.^39^ were not included in Table 3 and excluded from meta-analysis due to lack of clear reporting and the exclusion of patients with low adherence from their analysis.

#### Exploratory analyses concerning contributing factors

To explore factors contributing to variability in adherence, we conducted subgroup meta-analyses and meta-regression analyses. Specifically, we examined cancer type (solid tumors, hematological malignancies, mixed), treatment (TKI, chemotherapy, mixed), study duration (<6 months vs ≥6 months), and adherence measurement method (MPR, MEMS, PDC) as moderators in separate analyses.

Adherence was consistently lower in hematological malignancies compared to solid tumors (Cumulative *P* = .0006, Lower-bound *P* = .0008). Lower adherence was also observed when measured using PDC compared to MPR (Cumulative *P* < .0001, Lower-bound *P* = .046) or MEMS (Cumulative *P* = .001, Lower-bound *P* = .043). In contrast, differences related to treatment type were inconsistent between cumulative and lower-bound adherence analyses, with no clear overall pattern.

Cumulative adherence was lower in studies with longer duration (≥6 months) compared to shorter (<6 months) *P* = .0018, whereas no such difference was observed for lower-bound adherence (*P* = .40). The discrepancy may reflect that declines in adherence over time are not uniform across patients. Those who are initially non-adherent may progressively decline, while adherent patients generally maintain their adherence.

Although some moderators in the meta-regression were statistically significant, most of the between-study heterogeneity remained unexplained, indicating that additional factors may contribute to variability in adherence.

## Discussion

### Problems with adherence measuring methods

There have been several studies exploring the correlation between different methods of adherence measurement both in the oncological and non-oncological settings, several of which have shown a substantial difference in adherence rates using different measuring methods in the same population (see Table 4).

**Table 4.** Differences within the same population utilizing different adherence measuring methods.

Author	Adherence measuring method and reported adherence				
	MEMS	MPR/PDC	Pill-counts	Self-report	Doctors’ assessment
**Font et al.^28^**		74.7%		92%	94.7%
**Font et al.^29^**			67.9%	83%	100%
**Melis et al.^30^**		65%		82%	
**Schneider et al.^27^**		71%		89%	
**Waterhouse et al.^22^**	85.4%		92.1%	97.7%	
**Oberguggenberger et al.^32^**		85%		82%	92%
**Jasti et al.^23^**	64%		84%	77%	
**Ochagavía et al.^33^**			98%	86.9%	
**Noens et al.^31^**			90%	68% (BAAS) 95% (VAS)	

Abbreviations: MEMS = medical event monitoring systems, MPR = medication possession ratio, PDC = proportion of days covered, BAAS = Basel Assessment of Adherence Scale, VAS = Visual Analog Scale.

Considering this, additional research into the correlation between these methods and cautious assessment of adherence rates acquired by subjective measuring methods is recommended.

### Adherence in oncology

The different types of measurements listed above as “cumulative adherence,” “lower-bound-adherence,” “absolute adherence,” and “questionnaire-adherence” present challenges in obtaining a comprehensive overview of adherence. Different types of adherence measurement techniques further increase the difficulty in getting an overview of the “real” adherence to oral antineoplastic agents.

We argue that absolute adherence data contributes minimally to the academic discourse on adherence. Given the likelihood of occasional errors by patients over extended periods, sometimes spanning multiple years, the clinical impact of isolated missed doses is negligible. Furthermore, interventions designed to improve adherence should not be targeted solely at addressing sporadic lapses but rather directed towards populations exhibiting consistently lower adherence rates.

Several studies in this review showed excellent adherence, three of which, and some of the earliest works in this field, were published by Lee et al. in London 1992, 1993, and 1996^50^^,^^68^^,^^70^ reporting adherence rates between 93% and 100%. On the other hand, some studies show shockingly low adherence rates. Levine et al.^26^ and Kekäle et al.^47^ describes adherence rates of 16.8% and 21%, respectively. The reported adherence rates of all other included studies fall between these extremes.

To our knowledge, this review is the largest synthesis of studies on adherence to oral antineoplastic agents to date. Our meta-analysis combines data from 28 different studies and over 35 000 patients, providing estimated adherence rates that could help inform health care providers. The concept of lower-bound adherence translates well to clinical practice with the realization that approximately 3 out of 10 patients are at risk of significant undertreatment due to non-adherence. This should be taken into account in clinical practice, particularly in the event of treatment failure.

### Interventions to increase adherence

Available interventions have shown only limited effect in increasing adherence to oral antineoplastic therapy in a clinical meaningful way. In 2014, Nieuwlaat et al.^104^ published a Cochrane review of 182 RCTs investigating different types of interventions to increase adherence to medication, not cancer specific. The majority of studies had a high risk of bias. In the trials with the lowest risk of bias only a minority (5/17) demonstrated effect of the interventions tested, and no great effect was seen. Only complex and resource-heavy methods showed any effect. This is echoed in a meta-analysis by Conn et al.^105^ of 124 studies, including 19 348 patients on oral medication (not cancer-specific) in which different interventions were analyzed for their effect on adherence, with modest results.

In oncology, 2 studies examining interventions in populations with low adherence at baseline demonstrated a substantial effect. Levine et al.^26^ showed extremely poor adherence, ranging from 16.8% to 26.8% used urine-metabolite analysis. After exposure to rather simple interventions, such as disease and pill-taking education, adherence increased from below 20% to over 40%. In a study authored by Kekäle et al.,^47^ patients were randomized to either a control group or to a rather extensive intervention program, containing extra educational meetings with nurses and text message reminders. A clear benefit was shown for the intervention group, 51% versus 21%. This could indicate that low-adherence groups are responsive to interventions.

## Limitations

Search strategy, study selection, and data extraction were carried out by a single reviewer in a single database, which heightens the risk of bias and the likelihood that some relevant evidence was not identified. Additionally, the review was not preregistered in PROSPERO. The included studies were not subjected to any standardized quality assessment tool and are heterogenous in regard to measuring methods, adherence definitions, population, duration, and drugs. This introduces uncertainties that cannot be fully mitigated, and the pooled results should be interpreted with caution. Some studies were excluded from the meta-analysis due to missing data on standard deviation, which may have increased the risk of bias. Furthermore, quantitative adherence measures may not fully capture all variations, particularly if hospitalizations, dose adjustments, or temporary treatment interruptions are not considered.

## Conclusion

The definition of non-adherence and the measurement methods used across studies vary, rendering the comprehensive understanding of the subject challenging. This review and meta-analysis estimate the cumulative adherence to oral antineoplastic agents to be 84% (95% CI, 78–89) and lower-bound adherence at 71% (95% CI, 63–78). Adherence to oral antineoplastic agents deviates from full compliance across a wide variety of treatment regimens and tumor types, a potentially valuable insight for healthcare professionals in optimizing patient management.

## Supplementary Material

oyag168_Supplementary_Data

## Data Availability

The data underlying this article will be shared on reasonable request to the corresponding author.
