# Supplementary material for: Adherence to oral antineoplastic agents, an overview and meta-analysis
Source: Oncologist. 2026 Apr 30;31(6):oyag168. doi: 10.1093/oncolo/oyag168 (PMC13195812; doi:10.1093/oncolo/oyag168)
Supplement: oyag168_Supplementary_Data [file oyag168_supplementary_data.docx]

# Supplementary Material

**Supplementary Table S1.** Oral chemotherapy and targeted therapy available for use in Sweden 2024 (n=87)

| Abemaciklib  Abirateron  Afatinib  Akalabrutinib  Alektinib  Alpelisib  Axitinib  Bexaroten  Binimetinib  Bosutinib  Brigatinib  Busulfan  Ceritinib  Cyklofosfamidmonohydrat  Dabrafenib  Dakomitinib  Dasatinib  Enkorafenib  Entrektinib  Erlotinib  Estramustinnatriumfosfat  Etoposid | Everolimus  Fludarabin  Gefitinib  Gilteritinib  Hydroxikarbamid  Ibrutinib  Idarubicin  Idelalisib  Imatinib  Ixazomib  Kabozantinib  Kapecitabin  Klorambucil  Kobimetinib  Krizotinib  Lapatinib  Larotrektinib  Lenalidomid  Lenvatinib  Lomustin  Lorlatinib  Melfalan | Merkaptopurinmonohydrat  Metotrexat  Midostaurin  Mitotan  Neratinib  Nilotinib  Nintedanib  Niraparib  Olaparib  Osimertinib  Palbociklib  Panobinostat  Pazopanib  Pomalidomid  Ponatinib  Pralsetinib  Regorafenib  Ribociklib  Rukaparib  Ruxolitinib  Selperkatinib  Sirolimus | Sorafenib  Sotorasib  Sunitinib  Talazoparib  Talidomid  Tegafur, gimeracil, oteracil  Temozolomid  Tepotinib  Tioguanin  Tivozanib  Topotekan  Trametinib  Tretinoin  Trifluridin, tipiracilhydroklorid  Trofosfamid  Tukatinib  Vandetanib  Vemurafenib  Venetoklax  Vinorelbin  Zanubrutinib |
| --- | --- | --- | --- |

**
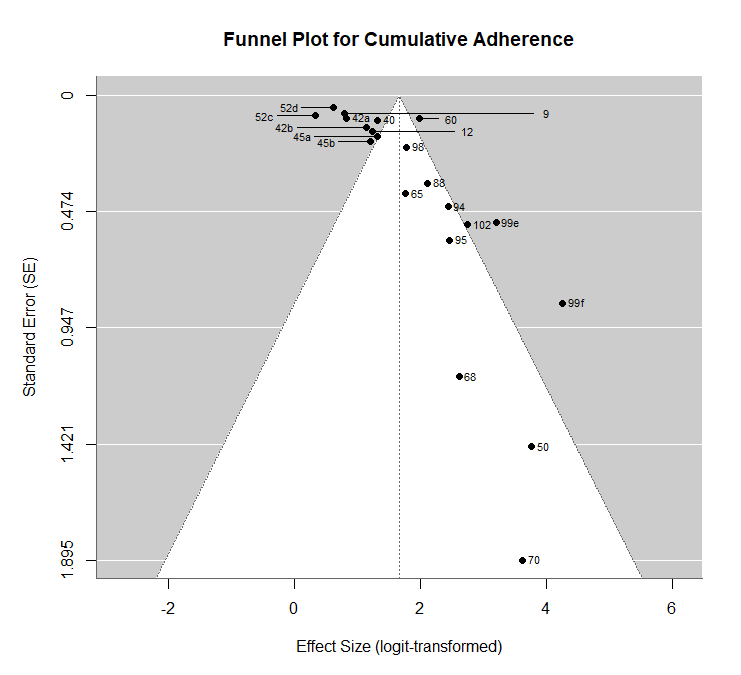
**

*a= Dasatinib, b= Nilotinib, c= Commercial, d= Medicare, e= Nurse-led, f= Pharmacist-led*

**Supplementary Figure S1.** Funnel plot for assessment of publication bias. Each point represents an individual study and the numbers correspond to the study reference numbers.

**
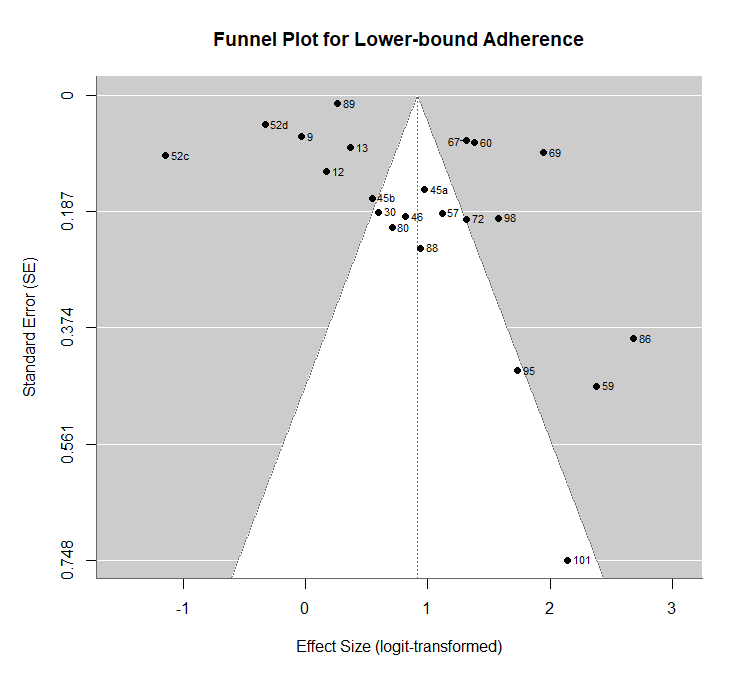
**

*a= Dasatinib, b= Nilotinib, c= Commercial, d= Medicare*

**Supplementary Figure S2.** Funnel plot assessment of publication bias. Each point represents an individual study and the numbers correspond to the study reference numbers.
